# Tunicates have a complex, highly dynamic TNF superfamily

**DOI:** 10.1371/journal.pone.0338039

**Published:** 2026-01-27

**Authors:** Ignacio Marín

**Affiliations:** Instituto de Biomedicina de Valencia, Consejo Superior de Investigaciones Científicas (IBV-CSIC), Valencia, Spain; Federal University of Rio de Janeiro, BRAZIL

## Abstract

Genes of the Tumor Necrosis Factor Superfamily (TNFSF) encode proteins with critical roles in cell signaling in animals, particularly in immunity and development. The evolution of the TNF superfamily remains poorly understood. This study demonstrates that tunicates possess a much more complex TNF superfamily than previously assumed. Some species have a large number of TNFSF genes, up to 14, due to frequent, independent tandem duplications. Significantly, this number exceeds that observed in many vertebrates (e.g., all characterized cyclostomes, as well as some birds and reptiles). As in vertebrates, the TNF superfamily of tunicates is capable of rapid evolutionary change. All 24 model tunicate species analyzed have different sets of TNFSF genes and even closely related species often have quite distinct TNF superfamilies. A comparison of tunicate and vertebrate data suggests that four TNFSF genes were present in the last common ancestor of both lineages. One of these genes was subsequently lost while the other three underwent independent duplications in early tunicate evolution. Six TNFSF genes were likely present in the common ancestor of all tunicates except perhaps appendicularians, for which data remain inconclusive. These results are consistent with the current model of how the TNFSF vertebrate genes emerged and evolved, except that whole-genome duplications played a significant role in expanding the family in vertebrates but not in tunicates. Transcription data obtained from *Ciona intestinalis* type A (*C. robusta*) indicate that several distantly related TNFSF genes share similar patterns of expression during development and in adults. Gene expression data are compatible with TNFSF genes having multiple roles, some of them in the innate immune system. Their involvement in immune defense may explain the rapid changes in TNFSF gene number observed in both tunicates and vertebrates.

## Introduction

In metazoans, one of the most important cell-signaling mechanisms involves the ligands of the Tumor Necrosis Factor superfamily (TNFSF) and their receptors, members of the TNF receptor superfamily (TNFRSF). Given their significance, the set of TNFSF and TNFRSF interacting pairs, often referred to as the TNFSF/TNFRSF system, has been carefully studied both functionally and evolutionarily. In vertebrates, it has critical roles in the adaptive immune system and also fundamental functions in apoptosis regulation, cell survival, proliferation and differentiation as well as in organogenesis [1–4]. With these varied tasks, it is unsurprising that several congenital human diseases are caused by mutations in TNFSF or TNFRSF genes [1,3,5]. In non-chordate invertebrates, roles in apoptosis, control of cell number and innate immunity have been described [6–13], suggesting that some functions of the TNFSF/TNFRSF system may be ancient. The evolution of the TNF and TNFR superfamilies has attracted considerable interest. One major focus has been to understand how the TNFSF/TNFRSF system emerged and diversified. Beyond its intrinsic evolutionary significance, this knowledge allows for translating functional results among species. Another line of research has explored the coevolution of the TNFSF and TNFRSF genes. Finally, other studies have examined the evolvability of the TNFSF/TNFRSF system, showing that it is high, given that related species often have considerably different sets of TNF ligands and receptors [14–21].

The TNFSF/TNFRSF system is restricted to metazoans and present in nearly all species. A few exceptions, such as *Caenorhabditis elegans* [22], lack both TNFSF and TNFRSF genes due to secondary losses. Mammals have large repertoires of both TNFSF and TNFRSF genes (e.g., 18 and 28, respectively, in humans). However, some non-mammalian species have even larger sets: the shark *Rhincodon typus* has 33 TNFSF genes, 25 are found in the sarcopterygian *Latimeria chalumnae*, at least 24 in the cephalochordate *Branchiostoma floridae* and, even more surprisingly, 23 TNFSF genes are found in a mollusk, *Crassostrea gigas* [18,20,21,23]. Similarly, 40 TNFRSF genes have been identified in the genome of the cnidarian *Acropora digitifera* [9] and 36 in *B. floridae* [23]. Given that simple invertebrates may have many genes while some vertebrates have reduced sets (e.g., only six to eleven genes are found in cyclostomes [21]), no obvious relationship exists between phenotypic complexity and the complexity of the TNFSF/TNFRSF system.

Many studies have analyzed the details of the evolution of the TNF superfamily in vertebrates, often focusing on particular groups [14,17,20,21,24–27]. Recently, I developed a comprehensive model for the evolution of this superfamily across all vertebrates, both cyclostomes and gnathostomes, based on the largest analysis performed to date, which included 23 model species [21]. In contrast, our understanding of how this superfamily evolved in other animals is very limited. Some studies have focused on non-vertebrate chordates, suggesting that tunicates have a few TNFSF genes, while cephalochordates have many [23,28–30]. However, the evolutionary analyses published so far that compare the genes of tunicates or cephalochordates with those of vertebrates [23,29–31] are both incomplete and inconclusive. This is not only due to the scarcity of sequence data available at the time, but also because the precise patterns of emergence of the vertebrate genes were still unknown, making impossible to propose a coherent model for the evolution of the TNF superfamily across all chordates.

This study aims to characterize the evolution of the TNF superfamily in tunicates with the goal of achieving a clearer understanding of the diversification of the superfamily across all chordates. Tunicates are the vertebrate sister group [32] and therefore are crucial to understand the early evolution of chordates. Comparing the tunicate and vertebrate TNFSF genes may provide significant information on the origin and evolutionary dynamics of the superfamily. Although, given their evolutionary proximity, it would be reasonable to expect similar sets of TNFSF genes in both lineages, the available data suggest that tunicates have only a few such genes. Five were detected in *Ciona intestinalis* [28,29] (more precisely, *C. intestinalis* type A, which is today also known as *Ciona robusta*; given that all the available sequence, gene and genome data remain annotated with the original *intestinalis* name, I will generally use it here to avoid confusions) and just two in the closely related species *Ciona savignyi* [30,31]. This conclusion will be tested here by expanding the analysis to other tunicate lineages. Furthermore, gene expression data from *C. intestinalis* will be explored to characterize how gene duplications have led to the functional diversification of the genes of this superfamily. As will be shown, the results are useful to both confirm and refine a model recently proposed, based on vertebrate data [21], offering new insights into the early evolution of the TNF superfamily.

## Materials and methods

### Nomenclature of the TNF superfamily

I will follow the conventions developed in my previous studies [20,21] for naming the genes of the superfamily in extant vertebrate species. In particular, the latter study classified all vertebrate genes into 35 orthology groups (orthogroups), with a total of 28 groups in gnathostomes and 7 in cyclostomes. Here, vertebrate genes will be named according to the orthogroup to which they belong. The precise relationships among these orthogroups were detailed in an evolutionary model [21], which will be used here as a guide. Ancient vertebrate genes will be named, following also the conventions developed in [21], as *TNFSF-Vx*, where “V” denotes they being pan-vertebrate genes, already present before the cyclostome/gnathostome divergence, and “x” is a number, which allows tracing their precise evolutionary relationships. The tunicate genes identified in this study will be named *TNFSF-Tx* (T: tunicate; x: gene-specific number). In some cases, an additional letter will be appended to the name to differentiate among recent duplicates. Correspondence with previously used names for *Ciona* TNFSF genes will be detailed along the text. Lastly, the most ancient chordate genes, present before the tunicate/vertebrate split, will be referred to as *TNFSF-Cx*, where “x” is again a number, chosen to both distinguish them and indicate their relationships.

### Sequence retrieval, alignments and phylogenetic analyses

The sequences of the TNFSF genes from 24 tunicate model species were obtained following the iterative search strategy already used in several of my previous works [14,21,33]. Briefly, TNFSF sequences of the selected tunicate species were identified through TBLASTN searches with default parameters against the nr, wgs, est, tsa, htgs and gss databases at the National Center for Biotechnology Information (NCBI) web page (http://blast.ncbi.nlm.nih.gov). The initial queries consisted of representative TNFSF protein sequences from all vertebrate orthogroups. Subsequently, the detected tunicate sequences were used as queries in similar searches, until no additional TNFSF sequences were detected. This approach was designed to prevent missing highly divergent sequences with low similarity to vertebrate genes. These searches allowed to detect all the TNFSF sequences of tunicate model species present in those databases at the time (December 2024). From these sequences, the highly conserved THD domain [5], the only region that can be reliably aligned in all TNFSF genes, was selected. Duplicated and partial sequences (i.e., those covering less than 90% of the THD domain) were then eliminated. The final dataset therefore includes only distinct and full-length or nearly complete sequences. Then, following the methods outlined in [21], the tunicate sequences thus obtained were aligned using twelve algorithms. Nine of them (FFTNS1, FFTNSF2, NWNS1, NWNS2, FFTNSI, NWNSI, GINSI, LINSI and EINSI) are implemented in the MAFFT program (version 7.5.0.5; [34]). The other three were ClustalX (Version 2.1; [35]), Clustal Omega (Version 1.2.0; [36]) and MUSCLE (version 5.1.0; [37]. In all cases, the default settings implemented in the programs were used. To perform FFTNSI, NWNSI, GINSI, LINSI and EINSI analyses, the user must specify the maximum number of refinement cycles of the alignment, which was set to 1000.

Maximum-likelihood (ML) analyses for tree construction were performed using the IQ-TREE program (version 2.3.6; [38]). The optimal substitution model for each of the 12 alignments was determined using ModelFinder [39]. Several studies have shown that it is convenient to vary the perturbation strengths (*pers* parameter) and to perform multiple tree searches to obtain optimal ML trees for a given alignment. Thus, following the recommendations of the IQ-TREE developers [40,41]. and as implemented in several of my previous works [14,21,33,42], the *pers* parameter was alternatively set to 0.2, 0.5 or 0.8, the number of replicates to stop the analysis (*nstop* parameter) was set to 500, and the number of independent runs (*runs* parameter) was set to 10. Consequently, a total of 36 different ML trees (from 12 different alignment algorithms, each one analyzed with 3 alternative perturbation strengths) were generated. In principle, the best tree would be the one with the highest ML value. However, since each protein substitution model implies a specific number of degrees of freedom and the alignments varied in length, it is more appropriate to compare the trees using the Bayesian Information Criterion (BIC; [43]), which takes into account these differences. Thus, the ML tree with the lowest BIC value was selected as the optimal one. After selecting this optimal tree, the corresponding alignment was compared with the alignments of gene pairs. When discrepancies were observed, the multiple sequence alignment was manually refined and the cycle of tree reconstruction repeated while the corrected alignments were producing improved, lower BIC values than the ones originally provided by the program. This led, as shown below, to small but still significant improvements of both ML and BIC values. Finally, to evaluate the topology of the tree, one thousand ultrafast bootstrap replicates (*bb* parameter; [44,45]) were performed to assess node support. Branches with bootstrap values of at least 95% were considered to have a significant statistical support [44].

### Synteny characterization

Local synteny data were collected to identify genes adjacent to the ones of the TNF superfamily in tunicate species. This information may complement the phylogenetic analyses: when very similar genes are identically located in two different species, it is most likely that they are orthologs. Given that most tunicate genomes are incompletely annotated, protein-coding genes were identified through comparison with a reference species, *Homo sapiens*. Around each TNFSF tunicate gene, 100-Kb genomic DNA fragments were selected and compared using BLASTX with default parametes against the human reference protein dataset at NCBI (refseq_protein database; accessed in January 2025), either until four genes were characterized both upstream and downstream of that TNFSF gene or the end of the available sequence was reached. The human genes showing the highest similarity in these BLASTX searches were considered the most likely orthologs of the tunicate genes. These inferred orthologies were used to establish local synteny similarities across tunicate species.

### Selection of sequences for tunicate/vertebrate comparisons

The comparison of tunicate and vertebrate TNFSF sequences in a phylogenetic context is complicated by the fact that some vertebrate TNFSF genes evolve at high rates [14,21]. To mitigate this problem, particular vertebrate orthogroups were selected according to a priori criteria. Given that the current model of vertebrate TNFSF genes evolution supports that all of them derived from four ancestral, pan-vertebrate genes called *TNFSF-V11*, *TNFSF-V12*, *TNFSF-V21* and *TNFSF-V22* [21], I selected all the orthogroups known to be present in the cyclostome ancestor (a total of six, whose origin from the four ancestral vertebrate genes is well-established; [21]) plus twelve gnathostome orthogroups, with three derived from each of the four ancestral genes. In some cases, more than three gnathostome orthogroups are known to descend from a given ancestral gene [14,21]. Then, using as a guide the most comprehensive tree available [21], the three orthogroups adjacent to the cyclostome groups derived from the same ancestor were chosen. This proximity indicates that those are the gnathostome genes with the slowest evolutionary rates among all candidates.

### Modelling the dynamics of the TNF superfamily in early chordate evolution

A model integrating all available data has been developed, following the strategy used to analyze vertebrate TNFSF genes [14,21]. In short, tree topology and synteny data are first considered to establish orthogroups and to identify closely related, recent paralogs. Then, the species phylogeny is considered to interpret the orthology/paralogy results, in order to infer how the genes emerged or became lost, attending to parsimony criteria.

### Expression data

Single-cell expression data during *Ciona intestinalis* type A (*C. robusta*) development were obtained from Cao *et al.* [46]. Results were visualized using the tools available on the corresponding website (https://singlecell.broadinstitute.org/single_cell/study/SCP454). Tissue-specific expression data in adults of the same species were obtained from Matsubara *et al.* [47].

## Results

### Diversity of the TNF superfamily in tunicates

A total of 168 TNFSF genes were identified across the 24 tunicate species analyzed, an average of seven genes per species. This was a surprising result, given that, as previously indicated (see Introduction), the number of genes hitherto reported in *Ciona* species was much smaller. The number of TNFSF sequences per species found here ranged from just one in the appendicularian *Oikopleura dioica* to 14 in *Boltenia villosa* and *Polycarpa mytiligera*, both of them stolidobranchian species. Significantly, seven genes were detected in *Ciona intestinalis* and twelve in *Ciona savignyi*, indicating that the previous analyses in both species were incomplete. Besides *Oikopleura*, which is known to have suffered an extreme genome reduction [48], the species with the fewest TNFSF genes were *Botryllus schlosseri* and *Pegea* sp., with only two sequences each. It was observed that species of the same genus often have quite different numbers of TNFSF genes, as occurs in *Ciona* (7 vs. 12 genes, as already mentioned), *Trididemnum* (3, 7 and 8 genes respectively, in three different species), *Halocynthia* (9 vs. 12 genes in two species) and *Styela* (10 vs. 13 genes in two different species). This is the first indication that the evolution of this superfamily in tunicates is highly dynamic, with gene duplications and losses occurring in relatively short periods of time.

**Fig 1:**
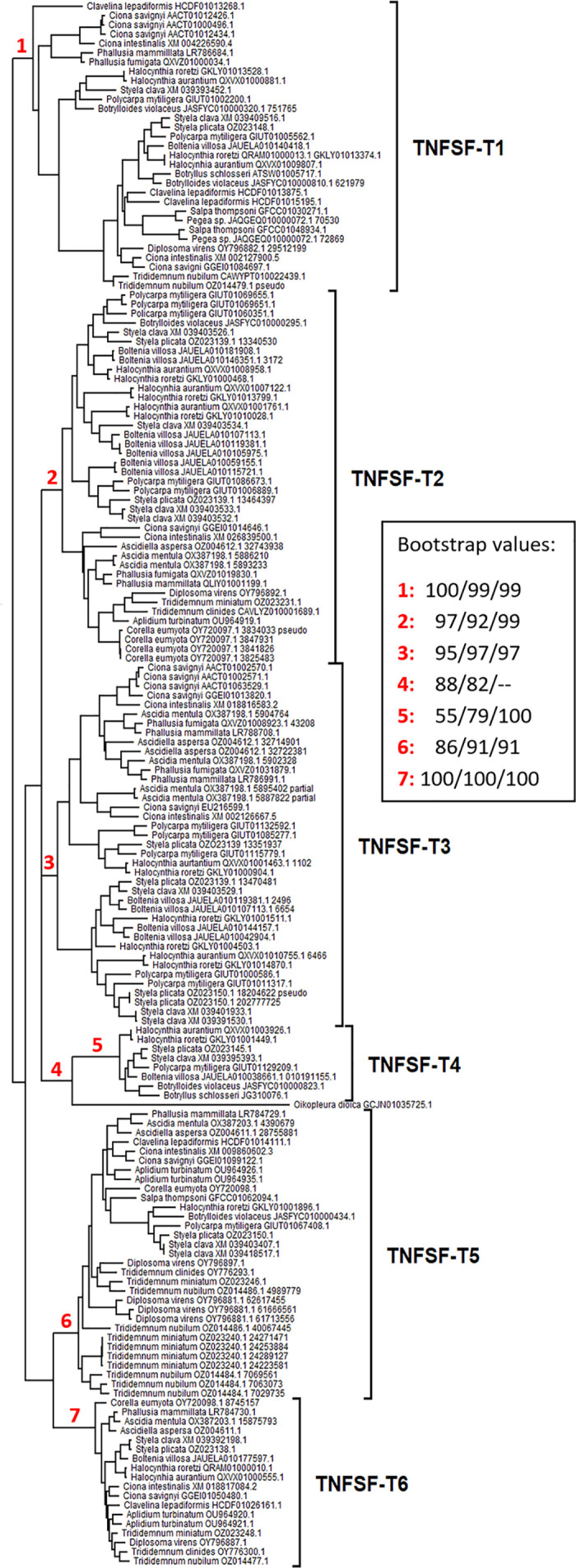
Summary of the ML trees obtained when analyzing tunicate TNFSF genes. For each sequence, the species name and accession number are indicated. When several sequences derived from the same contig, an additional number indicates where the TNFSF sequence starts. The topology shown corresponds to the optimal tree after manual correction of the alignment (see main text for details). The box displays the bootstrap values for seven key branches. From left to right, the values in the box correspond to: 1) the original CLUSTALX alignment; 2) the manually-corrected alignment; and, 3) this same corrected alignment once the highly divergent *Oikopleura* sequence is eliminated. Six highly-supported orthogroups are defined according to the topology of the tree and the patterns of presence/absence of genes in tunicate species. The final, manually-corrected alignment is detailed in the S1 File (see Supporting information).

The 168 tunicate TNFSF sequences were aligned and maximum-likelihood trees obtained, following the strategies described above. Among the trees generated by the twelve alignment strategies, the optimal one (i.e., the one with the lowest BIC) was obtained with the CLUSTALX alignment, the WAG + F + R6 substitution model and a perturbation strength of 0.5. This tree was built from an alignment with 209 columns and yielded a likelihood value of -lnL = 38394.583. Slight manual corrections, taking as guides the alignments of pairs of sequences, using the same evolutionary model and perturbation strength equal to 0.8, led to a more compact alignment (204 columns) with an improved score, -lnL = 38280.666. The corresponding BIC values were 78712.406 for the original alignment and just 78475.855 for the manually corrected one, confirming the latter as the better alignment. In any case, the topologies of both trees were nearly identical, with only minor changes in branches not relevant for the classification of the sequences into orthogroups and small variations in the bootstrap values. Fig 1 shows the final optimal ML tree, showing the bootstrap values of critical, internal branches in both the original and corrected analyses. Because it was observed that the single, highly divergent *Oikopleura dioica* sequence negatively affected some bootstrap values, especially lowering the value of branch 5 in Fig 1, an additional analysis was performed excluding that sequence. It was found that this elimination largely increased the bootstrap value for branch 5 but barely affected the values of the other critical branches (see data in Fig 1).

**Fig 2:**
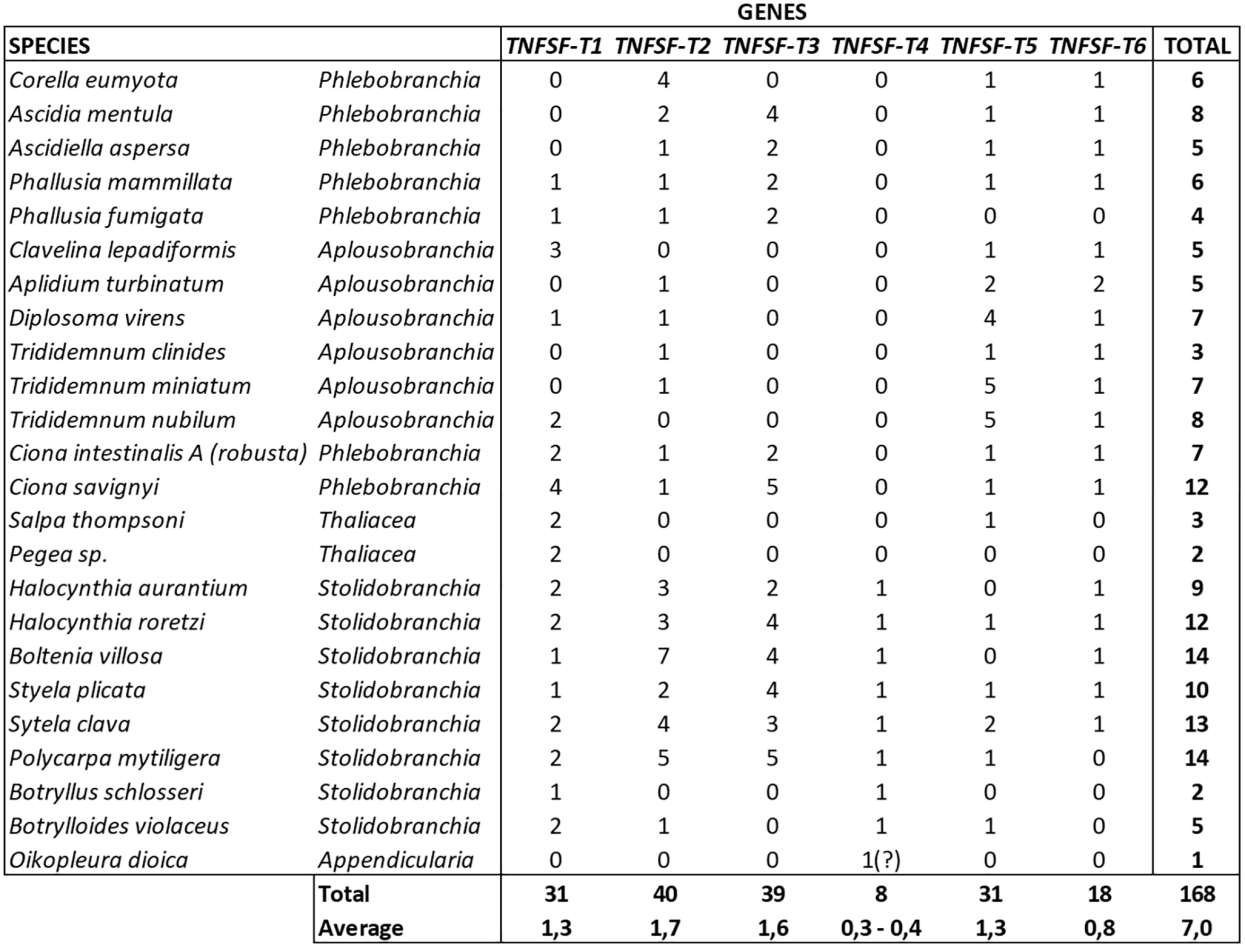
Number of genes per orthogroup in the model tunicates analyzed. The orthogroup to which the highly divergent *Oikopleura* sequence belongs is unclear. It is here assigned to TNFSF-T4, according to the results shown in Fig 1, but subsequent analyses did not confirm that association (see main text).

The tree topology revealed that all tunicate sequences can be grouped into a few highly-supported sets. After considering the distribution of the genes in each species, six orthogroups (TNFSF-T1 to -T6, see Fig 1) were defined. For five of them, significant (≥95%) bootstrap values were detected, once the *Oikopleura* sequence was excluded. For the other one (branch 6 in Fig 1), bootstrap value was just 91%. However, that branch includes species of all main tunicate taxa, in most cases with only one sequence per species, so it is clear that all of them belong to the same orthogroup. The relatively low bootstrap value observed is certainly due to the presence of eight divergent duplicates in species of the *Trididemnum* genus (see Fig 1), which may distort the topology obtained in some bootstrap replicates. Fig 2 summarizes the number of genes assigned to each orthogroup across the 24 tunicate species analyzed. Two of these genes have been functionally studied in previous works. Both of them, a *Ciona intestinalis* gene which was originally called “*CiTNFα*” [29,30,49,50] and a *Ciona savignyi* gene called “*CsTL*” [31], belong to the TNFSF-T3 orthogroup. They respectively correspond to the sequences with accession numbers XM_018816583.2 and EU216599.1 in Fig 1.

Molecular data suggested that, after the early divergence of appendicularians, the rest of tunicates split into two lineages about 390 million years ago [51–53]. One comprises thaliaceans, phlebobranchians and aplousobranchians, while the other includes the stolidobranchian species. All tunicate orthogroups except TNFSF-T4 are found in the genomes of species from both of these ancient branches, suggesting that at least five TNFSF genes were present in the ancestor of all tunicates except appendicularians. Actually, the minimal number of genes after the appendicularians separated from the rest of tunicates is six, given that two *TNFSF-T1* genes are observed in species belonging to both ancient branches of non-appendicularian tunicates (Fig 1). On the other hand, *TNFSF-T4* genes are found only in stolidobranchians, which suggests that this gene emerged more recently, in that group. With the available data, it is impossible to determine the number of TNFSF genes present in the appendicularian ancestor. The highly divergent *Oikopleura* sequence appears in Fig 1 close to *TNFSF-T4* genes; however, the bootstrap values for this association are not significant. Furthermore, it will be shown in the next section that the position of this sequence in the tree is unstable, changing when additional sequences are included in the analyses. Results in Fig 2 indicate that particular genes have been duplicated multiple times in some species and also suggest that gene losses are relatively common. Additional data are required to fully interpret the results shown in that figure in an evolutionary framework, which will be presented in the next sections.

### Synteny analyses of tunicate TNFSF genes

**Fig 3:**
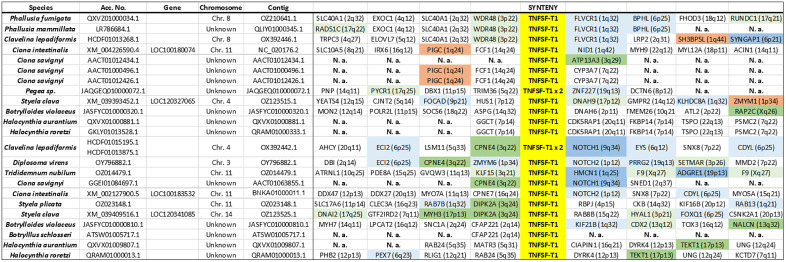
Synteny data for the region containing *TNFSF-T1* genes in different tunicate species. The names and chromosome positions (in parentheses) refer to the most likely human orthologs of the detected tunicate genes. Color codes indicate the degree of proximity respect to TNFSF genes in the human genome. Brown boxes indicate close proximity/adjacency to a TNFSF gene. Dark blue and dark green colors indicate that the corresponding human genes are located in regions that harbor TNFSF genes in our species, but are not adjacent to them. Light blue and light green colors indicate that a gene is found in a chromosome where TNFSF genes are present, but outside the regions that contain TNFSF genes. The blue shades refer to genes on human chromosomes 1, 6, 9 and 19, which contain regions derived from a particular ancestral chromosome. Green boxes indicate genes on human chromosomes 3, 13, 17 and X, which also have regions with a common origin (see details in the main text). N. a.: sequence data not available. Species for which no flanking genes could be identified are not included.

**Fig 4:**
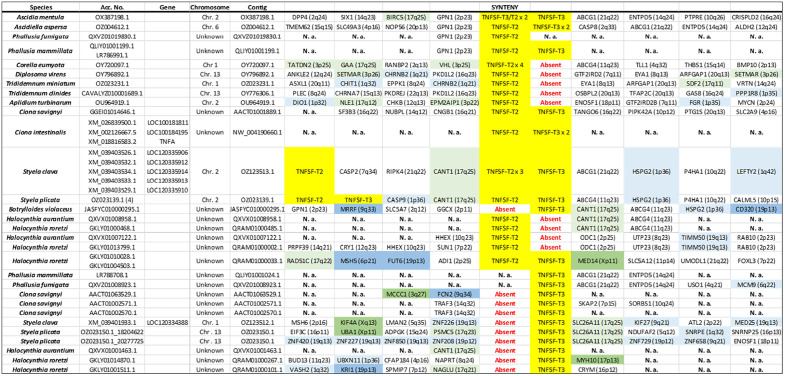
Synteny data for the regions around *TNFSF-T2* and *TNFSF-T3*, which are tandemly arranged in many species. Color codes and conventions as in Fig 3.

**Fig 5:**
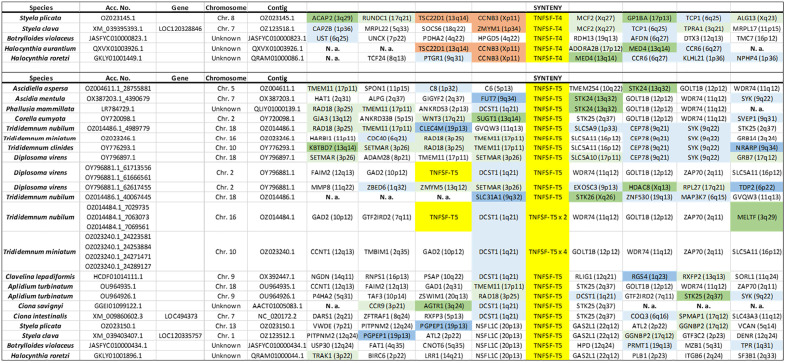
Synteny data for the regions which include *TNFSF-T4* (top) or *TNFSF-T5* (bottom).

**Fig 6:**
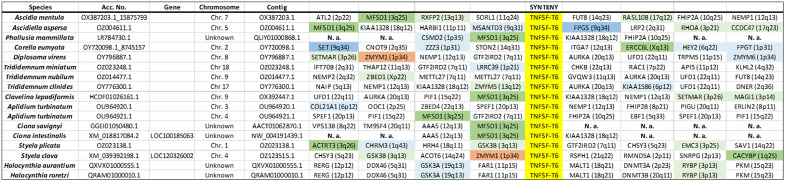
Local synteny data for the regions where tunicate *TNFSF-T6* genes are located.

Synteny data (summarized in Figs 3–6) provides additional insights to understand the evolution of the superfamily. A first finding that can be deduced from these datasets is that most new genes, in all species, emerged by tandem duplications whereas duplications associated to transpositions are uncommon. Also, whole-genome duplications do not have to be invoked to explain these results. A second fact is that *TNFSF-T2* and *TNFSF-T3* genes are located in tandem. This result, combined with the high similarity detected among the genes of these two orthogroups (see their positions in Fig 1), imply that both originated from a single ancestral gene. Similarly, *TNFSF-T5* and *TNFSF-T6* appear on the same chromosome in several species, probably because both arose from a tandem duplication, followed by inversions that separated them. This is again in good agreement with the results in Fig 1, which indicate a close relationship for these two genes. In all the other cases, the chromosomes in which the genes are located are different (see data in Figs 3–6). Another interesting finding is that TNFSF genes are generally flanked by unrelated genes, except in closely related species. This implies a high rate of chromosomal rearrangements, most commonly inversions, although, as already indicated, some transpositions or translocations may have also occurred. Several previous works already indicated that synteny is poorly conserved in tunicates [54–56]. Another conclusion that can be obtained from synteny data is that the duplicates detected correspond to different genes, and not allelic variants, given that they are surrounded by unrelated genes. There are only two exceptions, both in *C. savignyi*, where two slightly different TNFSF genes have apparently identical neighbors (see Figs 3 and 4). However, when the nucleotide sequences of these genes were compared, they were found to be quite distinct, suggesting that the patterns observed are due to very recent segmental duplications. A final result is related to the known positions of TNFSF genes in vertebrates. It is well established that TNFSF vertebrate genes are located on regions which descend from two ancestral chromosomes, present before vertebrates evolved (summarized in [21]). Whole-genome duplications (WGDs) have caused then to be now present in four chromosomes in cyclostomes (which suffered just one WGD; [57,58]) and, after two WGDs, in eight chromosomes in gnathostomes. In humans, those eight chromosomes are those numbered 1, 6, 9 and 19 (which contain common regions, derived from the ancestral chromosome called CGLM [57] or Pvc15 [58]) and 3, 13, 17 and X (similarly derived from the ancient chromosome CGLN [57], also called Pvc6 [58]). Since transpositions are rare, a logical prediction is that tunicate TNFSF genes should be generally surrounded by genes orthologous to those that in *Homo sapiens* have found on one of these eight chromosomes. Moreover, if genes have retained the same position since the tunicate/vertebrate divergence, we would expect identical adjacent genes in both lineages. Figs 3–6 provide data to test these predictions. It is observed that the number of genes close to vertebrate TNFSF genes whose orthologs are also found adjacent to the tunicate TNFSF genes (colored in brown in these figures) is low. This must be certainly due to the already mentioned high rate of genomic rearrangements in tunicates, which tend to separate adjacent genes. Nevertheless, the number of genes orthologous to genes located in the chromosomal regions where the vertebrate TNFSF genes are found (dark green, dark blue in these figures) is substantial, and, when the whole chromosomes are considered (adding to all the mentioned genes those colored in light green and light blue in the figures), there is a considerable number of positives, similarly distributed in all tunicate species. These findings strongly suggest that the tunicate and vertebrate TNFSF genes indeed derive from the same chromosomal regions, hence from common ancestral genes. The alternative possibility, that vertebrate and tunicate genes descend from totally different ancestral TNFSF genes, would predict genes flanking TNFSFs in both lineages to be totally unrelated, which is clearly not the case.

### Evolutionary relationships between tunicate and vertebrate TNFSF genes

**Fig 7:**
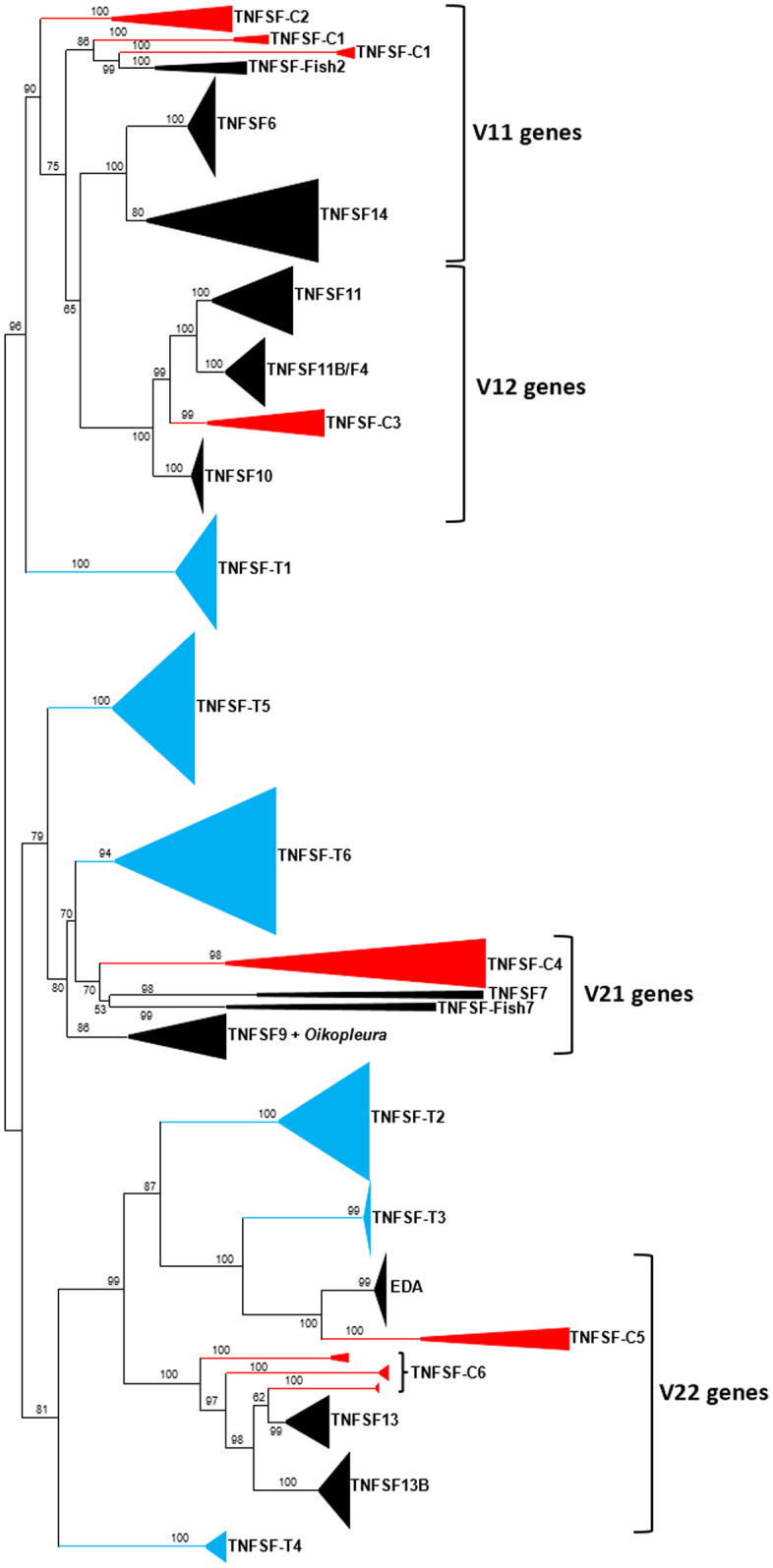
Optimal ML tree obtained when the tunicate sequences are analyzed together with selected vertebrate orthogroups. This tree was obtained after manually refining the alignment provided by the NWNSI routine in MAFFT, which was found to be the one that generated the best trees among the 12 alternative alignment programs (see Methods). Using the WAG + F + R6 model and a perturbation strength equal to 0.5, the ML value of the uncorrected tree was -lnL = 76249.295. ML analyses based on the corrected alignment, which had the same number of columns, were similarly performed. Using the same substitution model but with perturbation strength = 0.2, a slightly better tree was found (-lnL = 76210.959), which is the one shown here. The final alignment can be found in the supporting information of the paper (S2 File). Vertebrate genes fall into four distinct groups, each one derived from an ancestral gene, *TNFSF-V11* to *-V22*. Color coding: 1) blue: tunicate genes; 2) black: sequences of gnathostome vertebrates; and, 3) red: cyclostome vertebrate genes.

Although synteny provides significant indirect support, the hypothesis that vertebrate and tunicate TNFSF genes come from the same ancestral genes can be directly tested by comparing their sequences in a phylogenetic framework. Results are shown in Fig 7, which depicts the optimal (lowest BIC) tree obtained when the 168 TNFSF tunicate sequences were analyzed together with 218 vertebrate sequences, belonging to 6 cyclostome and 12 gnathostome orthogroups, selected according to the criteria described in the Material and Methods section.

Before analyzing in depth the results shown in Fig 7, it is significant to point out that the single *Oikopleura* sequence, which appeared close to *TNFSF-T4* genes in Fig 1, is located in this tree in a totally different place, within the vertebrate TNFSF9 orthogroup, very far away from *TNFSF-T4* and close to *TNFSF-T6* genes. This indicates that its position in the trees is unstable, so it is impossible to establish orthology relationships with genes of other species. Turning now to the general topology of the tree in Fig 7, a first key result is that the relative position of the vertebrate genes is identical to that found in my previous study [21]. The addition of the tunicate sequences did not affect the general disposition of the vertebrate genes, indicating that the global topology found both in [21] and here is robust. Now considering the position of the newly added tunicate genes, it turns out that there is a very good correspondence between the vertebrate and tunicate sequences. Thus, *TNFSF-T1* genes cluster in a highly supported branch (bootstrap = 96%) alongside with vertebrate genes derived from *TNFSF-V11* and *TNFSF-V12*. Also, *TNFSF-T5* and *TNFSF-T6* genes appear close to *TNFSF-V21*-derived genes. In this case, bootstrap support for the branch that includes them all is moderate (79%), but this may be explained by *TNFSF-T5* genes being very divergent from the rest, which creates ambiguities in the bootstrap replicates, diminishing both the value obtained for this branch and for the adjacent one, which puts together *TNFSF-T6* and the vertebrate *TNFSF-V21* genes (bootstrap = 80%; Fig 7). Finally, *TNFSF-T2*, *-T3* and *-T4* genes fall near *TNFSF-V22* genes, with the two first tunicate genes appearing in another highly supported branch (98% bootstrap value) together with vertebrate *EDA* and the rest of those genes (Fig 7). The association of *TNFSF-T4* genes with all the others is less well supported (81%) and indeed they appear in an intermediate position between V21- and V22-derived genes (Fig 7). This admits two explanations. The simplest one is that *TNFSF-T4* genes are close relatives of *TNFSF-T2* and *TNFSF-T3* genes but evolve very rapidly. The second is that *TNFSF-T4* is a distinct, tunicate-specific gene which has no counterpart in vertebrates and derive from a different, so far unknown, ancestral gene. Two facts argue against the latter hypothesis. First, *TNFSF-T4* is found only in stolidobranchians (Fig 2), which suggest a relatively recent origin. Second, *TNFSF-T4* genes are surrounded by genes whose orthologs are often found close to vertebrate TNFSF genes (Fig 5), which is hardly compatible with a totally independent origin.

### A model for the evolution of the TNFSF family in tunicates and vertebrates

The tree shown in Fig 7 indicates that only a few genes must be postulated as present in the common ancestor of tunicates and vertebrates. The topology of the tree may be interpreted as suggesting three ancestral genes (3G hypothesis), as follows: 1) the progenitor of *TNFSF-T1* and both V11 and V12 vertebrate genes; 2) the one from which *TNFSF-T5*, *TNFSF-T6* and vertebrate V21 genes derived; and, 3) the ancestor of *TNFSF-T2*, *TNFSF-T3* and *TNFSF-T4* plus vertebrate V22 genes. However, this hypothesis faces a significant problem when synteny data are considered. In vertebrates, V11 and V21 genes can be found in tandem and the same happens, in a different chromosome, for V12 and V22 genes. Given the high similarity of V11 and V12 genes and also that V21 are close to V22 genes in the trees (Fig 7; see also [21]) how these two tandems may have arisen from just three ancestral genes is very difficult to explain. An alternative hypothesis that easily explains all the data, including the synteny results, is that there were not three but four ancestral genes, distributed in two tandems, before the tunicate/vertebrate divergence (4G hypothesis). These four genes would exactly correspond to the already deduced vertebrate ancestral genes *TNFSF-V11*, *-V21*, -*V12* and *-V22*. In order to explain the results shown in Fig 7, it is sufficient to hypothesize that, after the tunicate and vertebrate lineages became separated, one of these genes was subsequently lost in tunicates. Results in Fig 7 are explained by the tunicate ortholog of vertebrate *TNFSF-V12* genes becoming lost and *TNFSF-T1* genes being fast-evolving relatives of vertebrate *TNFSF-V11* genes. Notice that, in Fig 7, *TNFSF-T1* genes are much closer to vertebrate *TNFSF-V11* genes than to *TNFSF-V12* genes. Significantly, the 4G hypothesis is further supported by published data from the third chordate lineage, cephalochordates, the sister group of both tunicates and vertebrates. Huang *et al.* [23] compared *Amphiouxus* genes with those in vertebrates, finding that some of them (which they called “TRAIL-like”) are very similar to vertebrate *TNFSF10* and *TNFSF11* (V12 genes) while others (“TNFA/FASLG-like” according to their nomenclature) were highly divergent genes found in their trees close to *TNFSF1*, *TNFSF2*, *TNFSF6* and *TNFSF14* (all of them V11 genes). The “TNFA/FASLG-like”/V11 genes most likely are the orthologs of the tunicate *TNFSF-T1* gene, which occupies the exact same position in the tree shown in Fig 7, while the “TRAIL-like”/V12 genes would correspond to the ones that the 4G hypothesis postulates as subsequently lost in tunicates.

**Fig 8:**
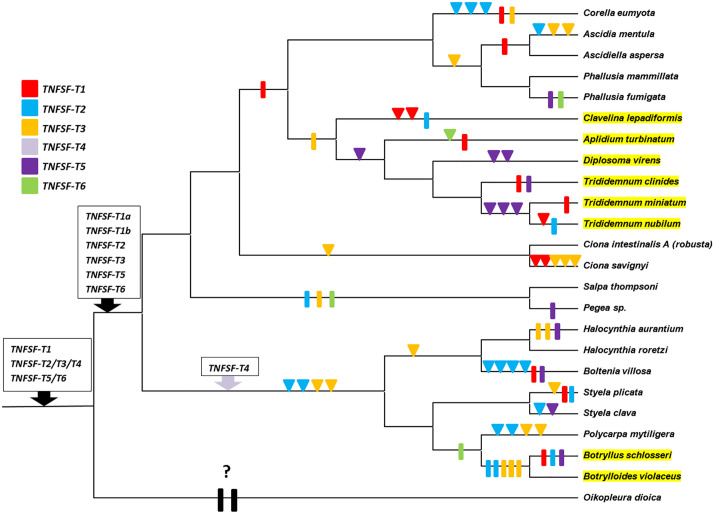
Most parsimonious model explaining the distribution of TNFSF genes in living tunicates. Colors are used to differentiate genes of each of the six orthogroups. Triangles indicate duplications and rectangles, gene losses. The topology of the tree is based on the known relationships among the model species analyzed [51–53]. Three genes are deduced to exist in the ancestor of all tunicates, which all duplicated to become six after the divergence of appendicularians. *TNFSF-T4* genes appeared relatively recently, within the stolidobranchian lineage. It was not possible to determine which genes were lost in appendicularians, given that the only sequence found in *Oikopleura* could not be reliably assigned to any orthogroup. Species names highlighted in yellow indicate colonial tunicates.

**Fig 9:**
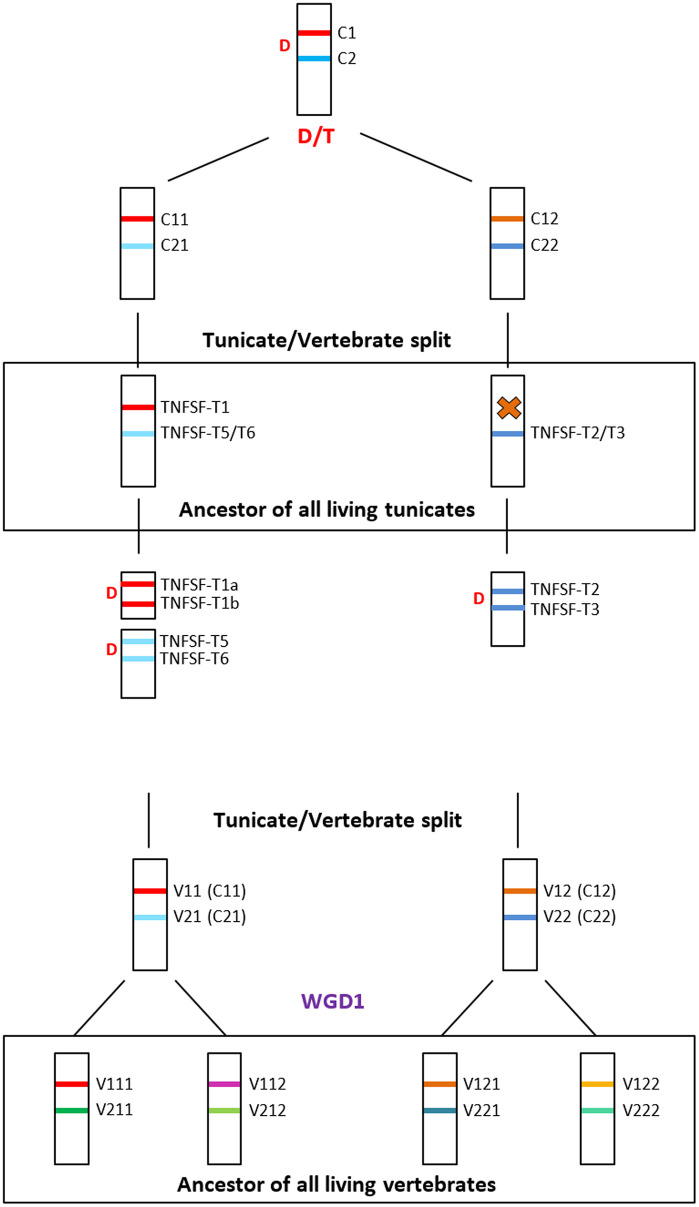
The 4G hypothesis explains all the data available in vertebrates and tunicates. Top panel: early evolution of the TNFSF genes and origin of the chordate ancestral genes and main tunicate orthogroups. A single gene became duplicated (D) in tandem. Later, a duplication plus transposition of that tandem (D/T) gave rise to the four ancestral chordate genes (*TNFSF-C11* to *-C22*). *TNFSF-C11* gave rise to tunicate *TNFSF-T1* genes, *TNF-C21* to *TNFSF-T5* and *-T6* and *TNFSF-C22* to *TNFSF-T2* and *TNFSF-T3*. One of those two last genes was much later duplicated again, in stolidobranchians, giving rise to *TNFSF-T4* (an event not depicted in this figure). *TNFSF-C12* was lost in the tunicate lineage. Bottom panel: evolution of the TNF superfamily in vertebrates after their divergence from tunicates. Descendants of the four ancestral chordate genes are found in living vertebrates. The ancestor of all vertebrates suffered a whole genome duplication (WGD1), so eight TNFSF genes were present in its genome. Much later, gnathostomes suffered a second whole-genome duplication (WGD2) with again duplicated the number of TNFSF genes (not shown in this figure). All the details of the subsequent evolution of the TNF superfamily in vertebrates can be found in [21].

Assuming that the 4G hypothesis is correct, it is simple to establish the most parsimonious explanation, in term of gene duplications and losses, for all the patterns described in Figs 1–7. Results are summarized in Fig 8. It is hypothesized that three genes were present in the common ancestor of all tunicates. Following the split of appendicularians, each of these three genes underwent duplication. These duplications gave rise to the ancestors of all orthogroups detected except for TNFSF-T4, which appeared later, in stolidobranchians (Fig 8). More recently, lineage-specific duplications and losses have occurred at a high frequency. Duplications have been concentrated in some lineages (as stolidobranchians, in which, in addition to the emergence of *TNFSF-T4*, both *TNFSF-T2* and *TNFSF-T3* have been amplified) and, as already deduced above, in particular species (compare, e.g., *Ciona savignyi*, *Polycarpa mytilligera* with their closest relatives). Interestingly, there is a general trend of the same genes being amplified several independent times in closely related lineages. On the other hand, some lineages (thaliaceans, *Halocynthia aurantium*, *Botryllus schlosseri*, etc.) have secondarily lost a significant number of genes (Fig 8). Duplications and losses have occurred at such frequencies that every tunicate species analyzed possesses a unique set of TNFSF genes. Significantly, exactly the same was observed for 23 model vertebrate species in my most recent analysis [21]. The conclusion is that the evolution of the TNF superfamily is highly dynamic in both vertebrates and tunicates. Finally, it can be deduced from Fig 8 that the transition from isolated to colonial modes of life did not affect the evolution of the TNF superfamily in any obvious, directional way. While colonial stolidobranchians (*Botryllus*, *Botrylloides*) suffered a very important loss of TNFSF genes, this did not occur in aplousobranchians, in which a slight tendency of gene number increase is observed. It is also noteworthy that both *Botryllus schlosseri* and *Salpa thompsoni* have two of the largest and more complex tunicate genomes, with at least 26000–27000 genes [59,60], yet their TNF superfamilies are among the smallest (Fig 2). Details of the 4G hypothesis are presented in Fig 9. Interested readers may find it useful to compare this figure with Figs 14 and 15 in [21], in which I already proposed the same 4G hypothesis to explain vertebrate TNFSF evolution and described the subsequent events in all vertebrate lineages, both cyclostomes and gnathostomes.

### Expression patterns of tunicate TNFSF genes

I have already shown that combining evolutionary and functional data provides valuable insights into the forces that drive the diversification of the TNF superfamily in vertebrates [21]. Data in tunicates are more limited, but still some significant results can be obtained. Two questions that may be explored using functional data are: 1) Do TNFSF genes, particularly the most recent duplicates, exhibit similar or different expression patterns? and, 2) Are the expression patterns of tunicate TNFSF genes consistent with the known functions of genes of this family in both tunicates and vertebrates?

**Fig 10:**
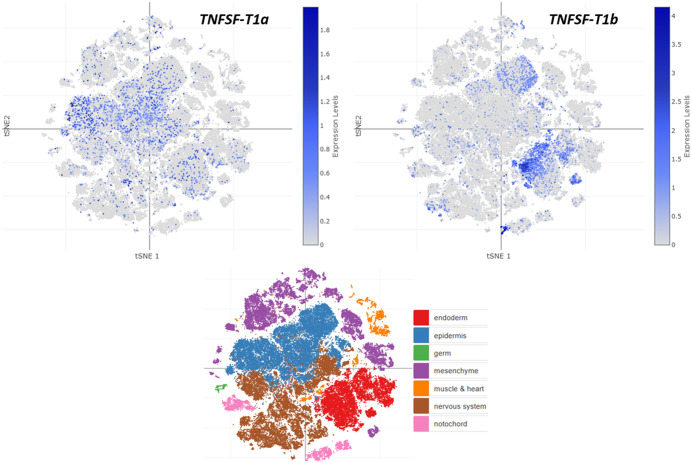
The very different expression patterns of *TNFSF-T1a* (left) and *TNFSF-T1b* (right) along *Ciona* development. The bottom panel indicates the regions that correspond to each tissue type in this two-dimensional representation [46].

**Fig 11:**
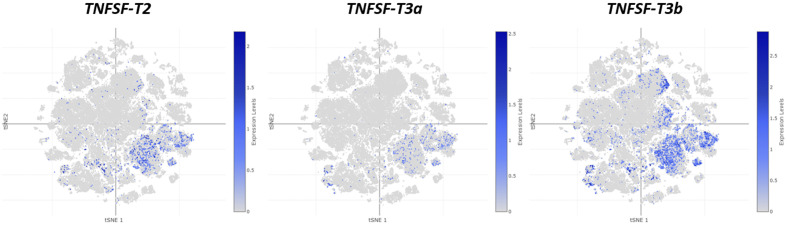
Overlapping developmental expression patterns of *Ciona TNFSF-T2*, *TNFSF-T3a* and *TNFSF-T3b* genes, which are also similar to those found for *TNFSF-T1b* (see Fig 10).

**Fig 12:**
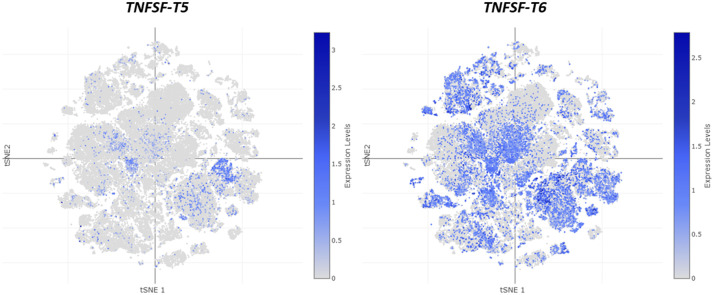
The expression patterns of the related genes *TNFSF-T5* and *TNFSF-T6* are qualitatively different. While *TNFSF-T5* has again a pattern quite similar to those of *TNFSF-T1*, *-T2* and both *-T3* genes (Figs 10, 11), *TNFSF-T6* has a singular, very broad expression pattern.

Figs 10–12 show global depictions of the patterns of expression of the TNFSF genes of *Ciona intestinalis* type A *(C. robusta)* during development, from early gastrula to larvae. The data, from Cao et al. [46], are presented as two-dimensional t-SNE plots which allow for a global appraisal of the expression levels of a given gene in all the *Ciona* cells analyzed (showed as small dots) at each developmental time and for each type of tissue (i.e., endoderm, epidermis, ectoderm, mesenchyme, muscle and heart, nervous system and notochord; see Fig 10, bottom panel, which indicates how the cells of each tissue are distributed in these images, according to [46]). This plot provides a general view of the similarity in expression among the genes, which may be completed by specific analyses to determine the precise meaning of each particular pattern. A good example is Fig 10 in which the patterns of the two *TNFSF-T1* genes of *C. intestinalis* are shown. It is obvious that the patterns are totally different, suggesting that the two genes may be performing unrelated tasks. When these patterns were analyzed in detail (data obtained from [46]), it was found that the first of the *TNFSF-T1* genes (which may be named *TNFSF-T1a* and corresponds to sequence XM_002127900.5) is mainly expressed throughout development in the epidermis, although it is also detected in other tissues, mostly endoderm and nervous system, especially during tailbud stages. On the contrary, the second gene (Acc. no. XM_0042265900.4, let us call it *TNFSF-T1b*) is predominantly expressed in endoderm in late tailbud stages and larvae, with some additional expression in epidermis and notochord, mostly also in the same, late developmental stages. Interestingly, when the pattern found for *TNFSF-T1b* is compared to those of other TNFSF genes, similar profiles are observed. It has been already shown that *TNFSF-T2* and the two *TNFSF-T3* genes present in *C. intestinalis* are relatively close evolutionary relatives (see Figs 1, 4, 7). Fig 11 indicates that the patterns of expression of these three genes closely resemble that of *TNFSF-T1b* (Fig 10; right panel). Expression is again concentrated on late developmental stages and in the endoderm. Detailed analyses confirmed what can be deduced from the figures, namely that one of the *TNFSF-T3* genes (which may be called *TNFSF-T3a* and corresponds to sequence XM_018816583.2; central panel in Fig 11) exhibits the most restricted expression pattern. The other two, in addition to the same main expression in endoderm along the late tailbud and larval stages, also display relevant levels of expression in other tissues (*TNFSF-T2* in nervous system throughout development and in notochord in gastrulae; *TNFSF-T3* XM_002126667.5, let us name it *TNFSF-T3b*, in nervous system and epidermis both in late tailbud and larval stages). Finally, Fig 12 shows the expression of the remaining two *C. intestinalis* genes, *TNFSF-T5* and *TNFSF-T6*. These two closely-related genes (Figs 1, 7) have totally different patterns of expression. *TNFSF-T5* is expressed only in a few cells of the epidermis in the gastrula and neurula stages and again, as already shown for *TNFSF-T1b*, *TNFSF-T2*, *TNFSF-T3a* and *TNFSF-T3b*, in the endoderm in the late tailbud stage and larvae. In contrast, *TNFSF-T6* has a broad expression pattern, being active in basically all tissues and all stages, with only a few exceptions (e.g., in late developmental stages, it is apparently active in only a very small number of cells of the epidermis or the notochord). This widespread pattern of expression, totally different from the one observed for the other TNFSF *Ciona* genes, does not appear to have a correlate in vertebrate species. *TNFSF-T6* is inferred to descend from the *TNFSF-C21* ancestral gene (Fig 9) but vertebrate genes derived from this ancestor and having such a broad pattern of expression have yet to be found. For example, the mammalian C21 (a.k.a. V21) genes *TNFSF4*, *TNFSF7*, *TNFSF8*, *TNFSF9* and *TNFSF18* play specific roles in particular cells of the adaptive immune system [4,61], which often leads to significantly correlated patterns of expression [21]. A very general role for any of these genes in early mammalian development is unlikely, as knockout mice for all of them are viable and fertile [62–67].

**Fig 13:**
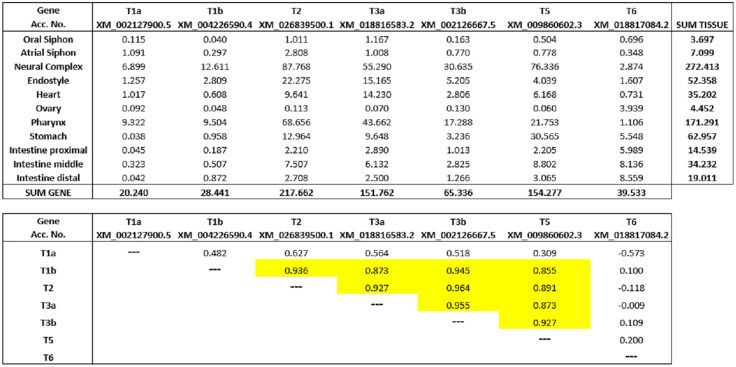
Expression levels of *Ciona* TNFSF genes in adult samples (data obtained from [47]). Top panel: raw expression levels, measured in RPKMs (reads per kilobase of transcript per million mapped reads). Bottom panel: Spearman’s correlation values. In yellow, significant correlations (p < 0.01; FDR-corrected).

Since the rudiments of adult organs are already present in developing tunicates before metamorphosis [68], expression patterns in adult *Ciona* tissues should often correlate with the ones found in previous stages. Matsubara *et al.* [47] obtained eleven different samples from the adult *Ciona*, corresponding to oral siphon, atrial siphon, neural complex, endostyle, heart, ovary, pharynx, stomach, and proximal, middle and distal intestine tissues. Results for the seven *Ciona* TNFSF genes are shown in Fig 13. The top panel displays expression levels, while the bottom panel indicates Spearman’s correlation coefficients, with the significant ones highlighted in yellow. Except for *TNFSF-T6*, the *Ciona* genes generally have their highest levels of expression in the neural tissues (derived from the ependymal cells and the neurons of the larvae [69]), as well as in the pharynx and endostyle, both endodermal derivates [68]. As already indicated, the cells of the endoderm and, to a lesser extent, those of the epidermis and nervous system, where the places were expression was most often detected for *TNFSF-T1b*, *TNFSF-T2*, *TNFSF-T3a*, *TNFSF-T3b* and *TNFSF-T5* during embryogenesis and in larval stages. When the adult expression patterns of these five genes are compared, it can be established that all of them significantly correlate (Fig 10, bottom panel). Thus, we may conclude that there are three different types of expression patterns for TNFSF genes in *Ciona* adults: 1) the five genes with similar patterns just mentioned; 2) *TNFSF-T1a*, which partially overlaps with those five (see the quite high positive but still not significant correlations in Fig 13); and, 3) *TNFSF-T6*, which displays a completely different pattern from the rest, with either very weak positive or even negative correlations with the other genes in adult tissues (Fig 13). Notably, these three adult patterns mirror the ones observed along development (Figs 10–12 and details above).

## Discussion

This article describes the emergence and evolution of the TNF superfamily genes in tunicates. The main conclusion is that this superfamily evolves in tunicate species in a very similar way as it does in vertebrates. In both groups, it shows both a general increase of complexity with time (although in particular lineages the number of genes may become secondarily reduced) and a rapid turnover, with both gene duplications and losses occurring very frequently. This leads to all species, even closely related ones, having different TNFSF gene sets (Figs 2, 8). This is exactly what was observed in vertebrates, when a similar number of species was examined [21]. Gene amplification has been especially pronounced in stolidobranchian species. It has been deduced that the ancestor of all stolidobranchians already had 11 TNFSF genes (Fig 8), which have been conserved or even further amplified in most extant species, reaching up to 14 genes in *Boltenia villosa* or *Polycarpa mytilligera* (Fig 2). The only exceptions are the colonial tunicates *Botryllus schlosseri* and *Botrylloides violaceus*, which secondarily lost most of the genes present in the stolidobranchian ancestor (Figs 2, 8). Outside the stolidobranchian lineage, only the phlebobranchian *Ciona savignyi* has a comparable number of genes, a total of twelve. However, in this case, most of the genes are very recent duplicates: five new genes emerged after the *C. savignyi*/*C. intestinalis* split (Fig 8).

Phylogenetic analyses indicate the presence of six tunicate orthogroups (Fig 1). It has been inferred that the common ancestor of all tunicates had three TNFSF genes and five were already present in the common ancestor of all tunicates except appendicularians (Fig 8). The sixth gene, *TNFSF-T4*, emerged in the stolidobranchian ancestor (Fig 8) most likely from either *TNFSF-T2* or *TNFSF-T3*, which are the most similar in sequence (Fig 1). The general reduction affecting the *Oikopleura dioica* genome [48] likely accounts for the disappearance of almost all TNFSF genes in this species. The single TNFSF sequence identified in *Oikopleura* could not be reliably assigned to any of the orthogroups (see details above). This is not unexpected, given the extreme sequence divergence found when most *Oikopleura* genes are analyzed; this species may be the fastest evolving metazoan [70]. With the exceptions of the thaliaceans *Salpa thompsoni* and *Pegea* sp. and the stolidobranchian *Botryllus schlosseri*, all the other species have retained most of the ancestral TNFSF genes. Only two of them, *TNFSF-T1* and *TNFSF-T3,* have been lost relatively often (Figs 2, 8), while the other are generally present. Particularly, *TNFSF-T4* is found in all stolidobranchians examined, without exception, and no duplication of this gene has been detected (Fig 2). *TNFSF-T2* is the gene with the highest average number of duplicates (Fig 2), largely due to multiple recent duplications occurred in particular stolidobranchian and phlebobranchian species (Fig 8). On the contrary, *TNFSF-T6* is the ancestral gene that has the minimal average of genes per species, with only one duplication observed against three independent losses (Fig 8). The fact that this gene is the one with the broader pattern of expression along embryonic development among all the *Ciona* TNFSF genes (Fig 12) may contribute to explain why it is difficult to accommodate either losses or increases in gene dosage.

Unlike in vertebrates, synteny has here provided limited insights about the relationships among TNFSF genes, due to the rapid change in gene order in tunicate species (Figs 3–6). The most significant results obtained are that most recent duplicates are found in tandem, that *TNFSF-T2* and *TNFSF-T3* genes are also located in tandem, implying a close relationship, and that *TNFSF-T5* and *TNFSF-T6* are found in several species in the same chromosome, indicating also a potential relationship. These results are consistent with the sequence similarity data summarized in the tree shown in Figs 1 and 7. Finally, the finding that many genes adjacent to the TNFSF genes in all tunicate species have close relatives, i.e., likely orthologs, in chromosomes where the human TNFSF genes are also found (see Figs 3–6) provides indirect evidence for all genes in vertebrates and tunicates emerging from common ancestors. The tree which combines vertebrate and tunicate sequences (Fig 7) has a topology that agrees well with that hypothesis. If the four TNFSF ancestral genes deduced for all vertebrate genes [21] were also the progenitors of the tunicate TNFSF genes, these last ones should be distributed in four regions of the tree and close to the corresponding four sets of vertebrate genes. These expected correspondences have been found in two cases (Fig 7), while, contrary to expectations, a single tunicate gene, *TNFSF-T1*, was found close to two different sets of vertebrate genes, those derived from the ancestral *TNFSF-V11* and *TNFSF-V12* genes (Fig 7). However, this pattern can be easily produced starting with four ancestral genes, if one of those genes was subsequently lost in all tunicates (as detailed in Fig 9). As already indicated, this 4G hypothesis is the only one that fits all the data obtained so far in both tunicates and vertebrates and moreover has additional support from an analysis of the TNF superfamily in cephalochordates [23].

Expression data for the seven TNFSF genes present in *Ciona intestinalis* type A (*C. robusta*) reveal three qualitatively different patterns. Five genes, *TNFSF-T1b*, *TNFSF-T2*, *TNFSF-T3a*, *TNFSF-T3b* and *TNFSF-T5* display similar expression profiles both along development and in adults (see Results section). In contrast, the other two, *TNFSF-T1a* and *TNFSF-T6* show distinctive configurations. Highly correlated patterns of expression have been observed also for many vertebrate TNFSF genes and evidence for these correlations implying that they are performing related tasks in the same processes, most significantly in cell-to-cell communication among cells of the adaptive immune system, has been obtained [21]. This suggests the hypothesis that the correlated tunicate TNFSF genes also participate in the same processes. Functional data are currently available for only one of the *C. intestinalis* genes, *TNFSF-T3a*, which was originally called “*CiTNFα*” due to it having some similarity to human *TNFSF2* (a. k. a. *TNF, TNFα*) [29]. However, data obtained here demonstrate that this similarity is not significant. *TNFSF2* is a V11 gene [21], while tunicate *TNFSF-T3* genes are close relatives to vertebrate V22 genes (Fig 7). Interestingly, vertebrate V22 genes are quite peculiar in that their involvement in the adaptive immune system is limited. For instance, the *EDA* gene is broadly expressed and mutations in the gene lead to multiple developmental anomalies (abnormal skin, teeth, some glands) in both mice and humans [71–73]. Also, it shows no correlation of expression with any of the other TNFSF genes, most of them highly expressed in cells involved in adaptive immunity [21]. The other two mammalian V22 genes, *TNFSF13* and *TNFSF13B*, show only significant coexpression with a few TNFSF genes and are highly expressed mainly in macrophages and related cells (such as monocytes, Langerhans or Hofbauer cells), but not in lymphocytes or NK cells [21]. It is well known that macrophage-like cells are ancient, being present in animals without an adaptive immune system. All these results point towards TNFSF-V22-derived genes having retained in mammals, and potentially in other vertebrates, primitive roles unrelated to the recently evolved adaptive immune system. In this context, it is significant that the V22-related *C. intestinalis TNFSF-T3a* gene has been shown to be upregulated in the adult pharynx after treatments that produce local inflammation, activating the ascidian innate immune system [29,50,74]. The same was found in several tissues of *Ciona savignyi* for one of its five *TNFSF-T3* genes, originally called “*CsTL*” [31], which is orthologous to *Ciona intestinalis TNFSF-T3b* (Fig 1; see above). A stress regime involving hypoxia and starvation also activates *TNFSF-T3a* in *C. intestinalis* [74]. The known roles of the pharynx and endostyle in innate immunity [75] agree well with the fact that *TNFSF-T3 Ciona* genes are highly expressed in those tissues (Fig 13). The high expression in endodermic cells throughout development (Fig 11 and see details in the text) may be related, given that both pharynx and endostyle are endoderm derivates [68,76]. This naturally leads to the hypothesis that not only *TNFSF-T3a* and *TNFSF-T3b* but also the other three genes that show high correlation of expression with those two, i.e., *TNFSF-T1b*, *TNFSF-T2* and *TNFSF-T5*, may be all performing related functions in the innate immune system of tunicates, perhaps providing distinct signals to coordinate the rapid responses required to cope with infections or other types of damage. Significantly, a similar involvement for cephalochordate TNFSF genes in innate immunity, which may be the main force explaining the expansion of the superfamily in that lineage, has been previously suggested [23]. The general involvement of the TNFSF genes in the immune defense systems may underlie many of the dynamic changes observed in chordates. Different organisms, even if evolutionarily closely related, may be exposed to distinct stressors (specific types of physical damage or harmful stimuli, different pathogens, etc.). The TNFSF/TNFRSF system may therefore need to be continuously fine-tuned to cope with these shifting challenges. Gene duplications followed by functional diversification of the duplicates, as well as occasional gene losses when particular genes are no longer useful, may be the most straightforward way to refine the system. Now that the full complexity of the tunicate TNF superfamily has been characterized, this functional hypothesis can be readily tested in model species. Other significant results found here may also elicit new research lines. For example, the finding of a high level of expression of the five correlated *Ciona* TNFSF genes in the adult neural complex, despite their limited expression in nervous system cells along development (see above), may indicate an additional, adult-specific function. An attractive hypothesis is that these five genes have a role in signaling to recruit cells required for wound healing or tissue regeneration in adult tunicates [77,78]. Additional functions may be expected to be found when analyzing the two other *Ciona* genes, *TNFSF-T1a* and *TNFSF-T6*. Their expression patterns suggest that they may have roles unrelated to those performed by the quintet of correlated genes. Finally, functional analyses of tunicates with very complex TNF sets may no doubt unearth additional novel results, contributing to a better understanding of the functions of this superfamily both in tunicates and in other chordates.

## Supporting information

S1 File Optimal alignment corresponding to Fig 1. (TXT)

S2 File Optimal alignment corresponding to Fig 7. (TXT)
